# Relating ocean temperatures to frontal ablation rates at Svalbard tidewater glaciers: Insights from glacier proximal datasets

**DOI:** 10.1038/s41598-019-45077-3

**Published:** 2019-07-01

**Authors:** Felicity A. Holmes, Nina Kirchner, Jakob Kuttenkeuler, Jari Krützfeldt, Riko Noormets

**Affiliations:** 1 Glaciology and Geomorphology Unit, Department of Physical Geography, Stockholm University, 106 91, Stockholm, Sweden; 2 Bolin Centre for Climate Research, Stockholm University, 106 91 Stockholm, Sweden; 3 Department of Arctic Geology, University Centre in Svalbard, PO-box 156 N-9171, Longyearbyen, Svalbard Norway; 4 Centre for Naval Architecture, KTH Royal Institute of Technology, SE-100 44 Stockholm, Sweden

**Keywords:** Cryospheric science, Ocean sciences, Environmental sciences

## Abstract

Fjord-terminating glaciers in Svalbard lose mass through submarine melt and calving (collectively: frontal ablation), and surface melt. With the recently observed Atlantification of water masses in the Barents Sea, warmer waters enter these fjords and may reach glacier fronts, where their role in accelerating frontal ablation remains insufficiently understood. Here, the impact of ocean temperatures on frontal ablation at two glaciers is assessed using time series of water temperature at depth, analysed alongside meteorological and glaciological variables. Ocean temperatures at depth are harvested at distances of 1 km from the calving fronts of the glaciers Kronebreen and Tunabreen, western Svalbard, from 2016 to 2017. We find ocean temperature at depth to control c. 50% of frontal ablation, making it the most important factor. However, its absolute importance is considerably less than found by a 2013–2014 study, where temperatures were sampled much further away from the glaciers. In light of evidence that accelerating levels of global mass loss from marine terminating glaciers are being driven by frontal ablation, our findings illustrate the importance of sampling calving front proximal water masses.

## Introduction

The rates of frontal ablation at tidewater glaciers are a key uncertainty in future sea level rise projections^1^, but direct observations of calving and submarine melt, two processes collectively referred to as frontal ablation, are difficult to obtain. Estimates can be derived from oceanographic data, but this is spatially and temporally scarce along marine ice margins. For sites in Svalbard and Greenland, previous studies have found that ocean temperatures at depth exert a primary control on the frontal ablation rate of marine-terminating glaciers^2,3^. As perturbations at glacier termini have been observed to lead to glacier thinning, acceleration, and further retreat, with these dynamic impacts extending upstream of the terminus^4–7^, variations in frontal ablation processes in response to warming ocean waters are of importance for the mass balance and dynamics of tidewater glaciers. However, as water masses may be transformed as they journey into fjords and towards glacier termini as a result of regional oceanographic, atmospheric, and hydrological processes^8–12^, it is important to interpret findings concerning possible major drivers of frontal ablation with care - especially in the absence of local measurements.

In the wake of the recently observed atlantification of the Arctic Ocean and the Barents Sea^13–15^ some of Svalbard’s tidewater glaciers have become exposed to increasingly warmer waters at their marine ice fronts^14^, rendering them ideal sites for investigating controls on frontal ablation. However, local processes at the ice-ocean interface add complexity. Subglacial discharge may feed plumes developing along the calving front which turbulently entrain warm water and bring it into direct contact with the ice front, promoting submarine melt^16^ and causing undercutting^17^. Additionally, bathymetry may act to modulate or exacerbate changes, as well as having the potential to block or permit the intrusion of warm water into fjord systems^10,18–20^. There is therefore a particular need for studies that account for the temperatures of the water masses actually in contact with the glacier front, by sampling oceanographic data from sites sufficiently close to the glacier. Failure to do so increases the potential for under- or over-estimates of ocean-temperature derived rates of frontal ablation.

**Figure 1:**
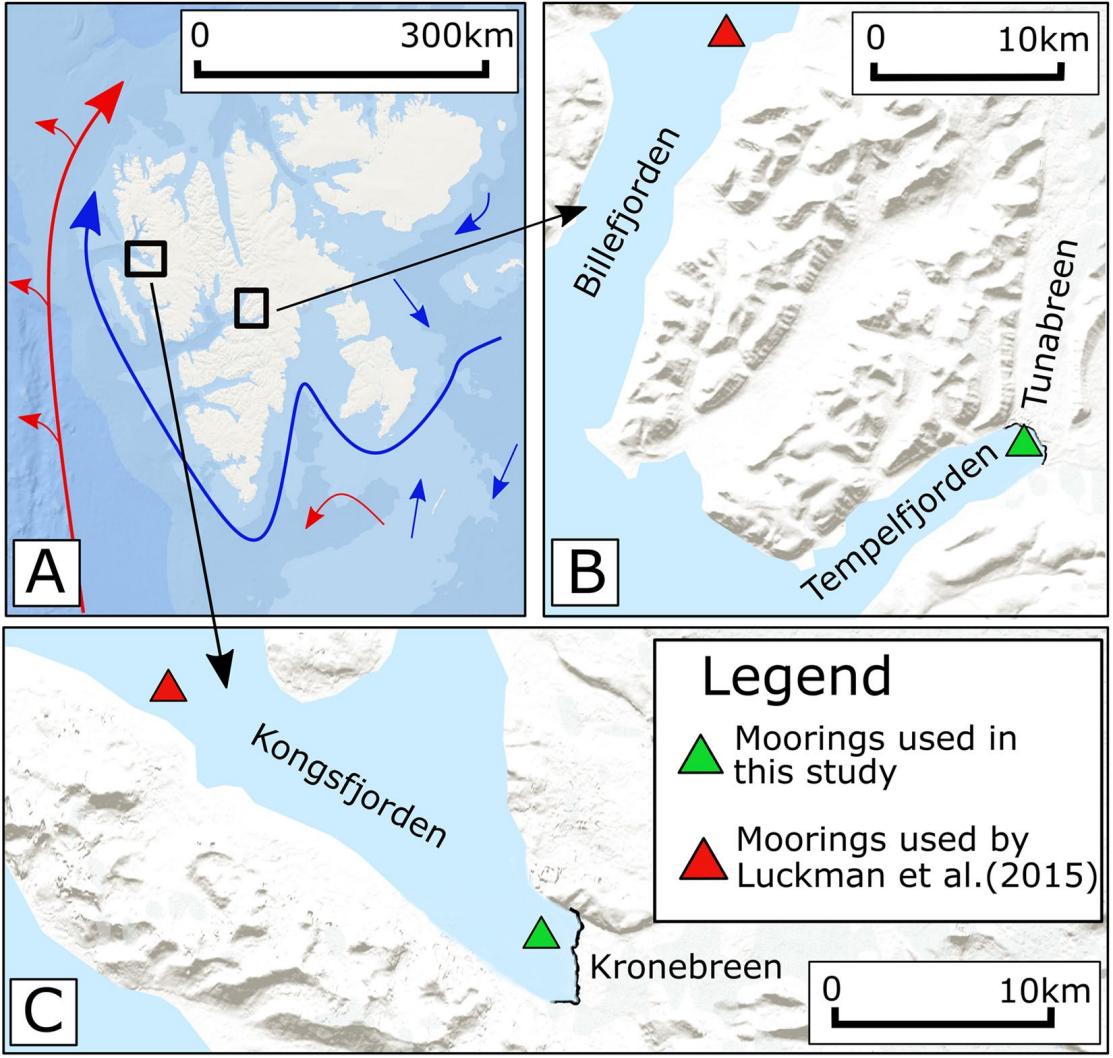
(**A**) Map of Svalbard showing the location of the Kronebreen and Tunabreen, as well as the position of ocean currents adapted from Sundfjord *et al*.^9^: West Spitsbergen Current - red; cold currents- blue. (**B**) The study site in Tempelfjorden, showing the location of the moorings used in this study, and by a 2013–2014 study^2^. (**C**) The study site in Kongsfjorden, showing the location of the moorings used in this study, and by a 2013–2014 study^2^. Background images for all panels from Source: Esri, DigitalGlobe, GeoEye, Earthstar Geographics, CNES/Airbus DS, USDA, USGS, AeroGRID, IGN, and the GIS User Community. Panel A created using Inkscape version 0.92.3 (https://inkscape.org/release/inkscape-0.92.3/), and panels B and C created using QGIS version 3.0-Girona (https://qgis.org/en/site/forusers/download.html)^38^.

We present a 376 day time series of water temperature, collected by a LoTUS buoy moored at 67 *m* water depth in Kongsfjorden, ca 1 *km* from the calving front of Kronebreen, western Svalbard to assess drivers of frontal ablation between 25 August 2016 and 4 September 2017 (see Fig. 1). For the Tunabreen study area, a 232 day (2 September 2016 until 21 April 2017) time series of water temperature, collected from another LoTUS buoy at a depth of 19 *m* in Tempelfjorden and at a distance of ~600 *m* to the front of Tunabreen is instead used (see Fig. 1). We compare our findings to results reported for both glaciers during an earlier observation period (2013–2014), where ocean temperatures at depth were sampled much further away from the glacier fronts. Specifically, ocean temperatures were collected from a mooring 18 *km* away from Kronebreen and, for Tunabreen, from a branch of Isfjorden distinct from that which Tunabreen calves into^2^. Results relating to Kronebreen will also be compared to a calculated value for submarine melt.

## Results

### Characterization of fjords and glaciers studied

**Figure 2:**
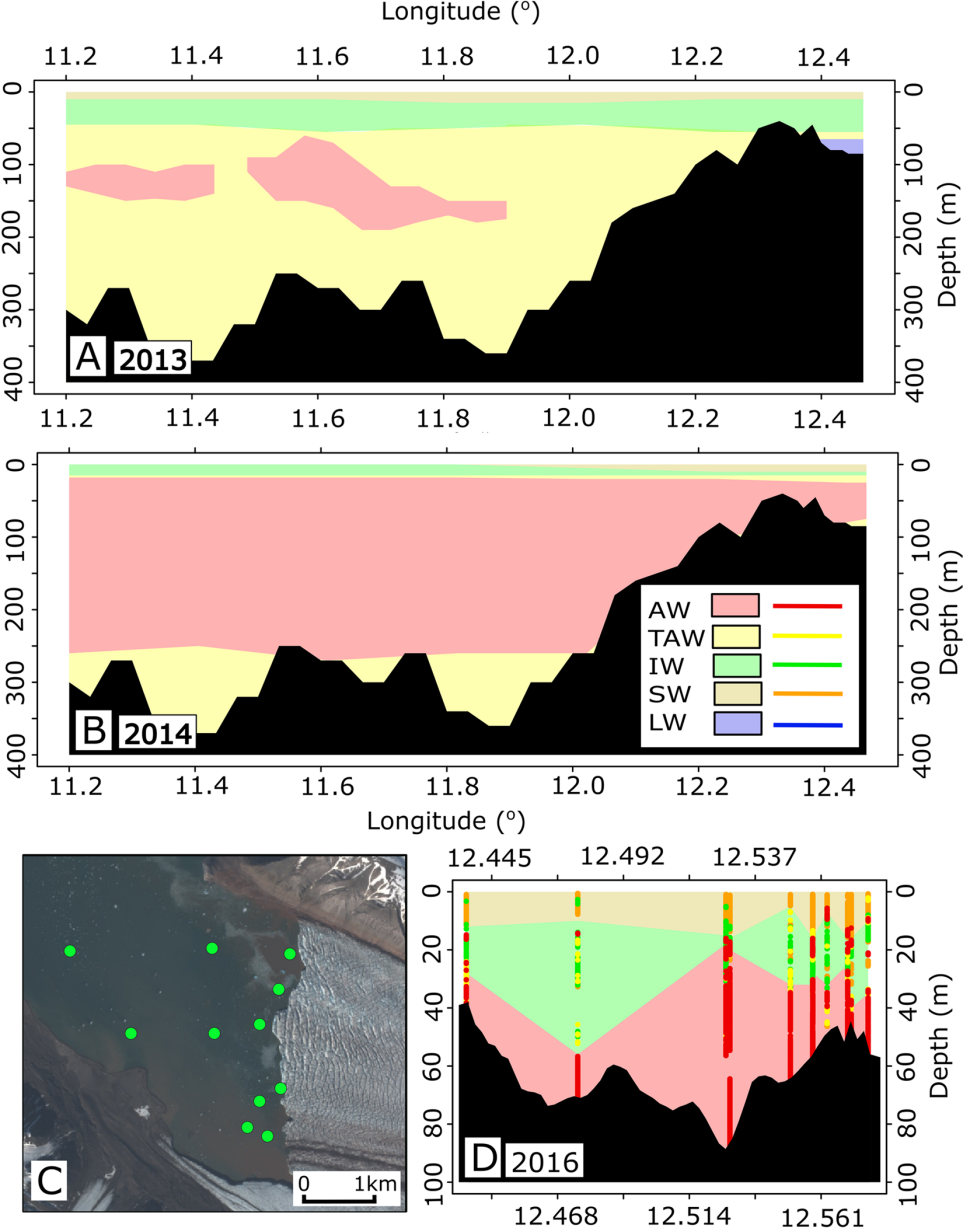
Water masses in Kongsfjorden during 2013, 2014, and 2016. Panels A and B (2013 and 2014) are adapted from the results reported by Prominska *et al*.^23^. Panel C shows the locations of the SVP profiles taken during the course of this study (green dots) during August 2016. Panel D is a conceptual sketch of the water masses present in the inner fjord, based on a number of profiles (shown in bold) taken at the locations shown in panel C. Note the different depth and longitude scales in panel D compared by panels A and B. In the legend, Atlantic water is denoted AW, Transformed Atlantic water is TAW, Intermediate water is IW, Surface water is SW, and Local water is LW. More detail on these classifications in available in the methods. The background image in panel C is Copernicus Sentinel data (2016) downloaded from the Copernicus Open Access Hub (https://scihub.copernicus.eu/). Terms of use available at https://scihub.copernicus.eu/twiki/do/view/SciHubWebPortal/TermsConditions. Panels A, B, and D were created in R^36^.

Two glacier-fjord systems are studied here, the first of which is the Kronebreen/Kongsfjorden system, located on the western coast of Svalbard (see Fig. 1). Kronebreen, at 78.8°N, is a fast-flowing, polythermal, surge-type glacier currently in a quiescent phase^21^. Kongsfjorden is 22  *km* long and between 4 and 12 *km* wide^22^, which is sufficient for its circulation to be influenced by the Coriolis force^10^. In terms of bathymetry, it does not have a pronounced sill and is thus susceptible to intrusions of water from outside of the fjord^23^. Its physical environment varies seasonally as a result of variations in the balance of warm Atlantic waters and cold Arctic waters, the volume of glacial melt^11^, and as a result of changes in wind direction which is mostly down-fjord during winter and up-fjord during summer^10^. Recently, a general increase in the presence of Atlantic water (AW) in Kongsfjorden has been reported^14^. Sound velocity profiles (SVP) taken at various calving front proximal sites between 23 and 26 August 2016 show that 55% of observations fall into the AW classification range, suggesting a high proportion of warm water in the inner part of Kongsfjorden (see Methods). This data also shows that the warmest water temperatures occurred at depths of 20–25 *m*, evidenced in Supplementary Material Section S1. A previous paper by Prominska *et al*.^23^ presented plots of the water masses present in Kongfjorden for 12 different summers between 2001 and 2015, in which AW was present during 9 of those summers. However, the AW was only found East of 12.2°E (in the inner fjord) during one of those years (2014). Adaptations of two of these water mass plots are presented in Fig. 2, corresponding to the years 2013 and 2014.

The second area of interest is the Tunabreen/Tempelfjorden system (see Fig. 1 for location). Tunabreen is a surge-type glacier which has experienced three surges since the Little Ice Age^24^. Recent observations of Tunabreen have found low velocities (less than 1 *md*^−1^ during winter) and a trend of retreat interspersed with small winter re-advances^24^. Tempelfjorden is part of the Isfjorden system and is around 14 *km* long with a width of between 3 and 5 *km*. The maximum water depth in Isfjorden is 110 *m*^24^.

### Frontal ablation rate and ocean temperatures at depth

Frontal ablation rates are derived from satellite data products (see Methods) and are calculated from Eq. 1 ^2^.

1 $$\dot{a}={U}_{T}-\frac{dl}{dt}.$$

**Figure 3:**
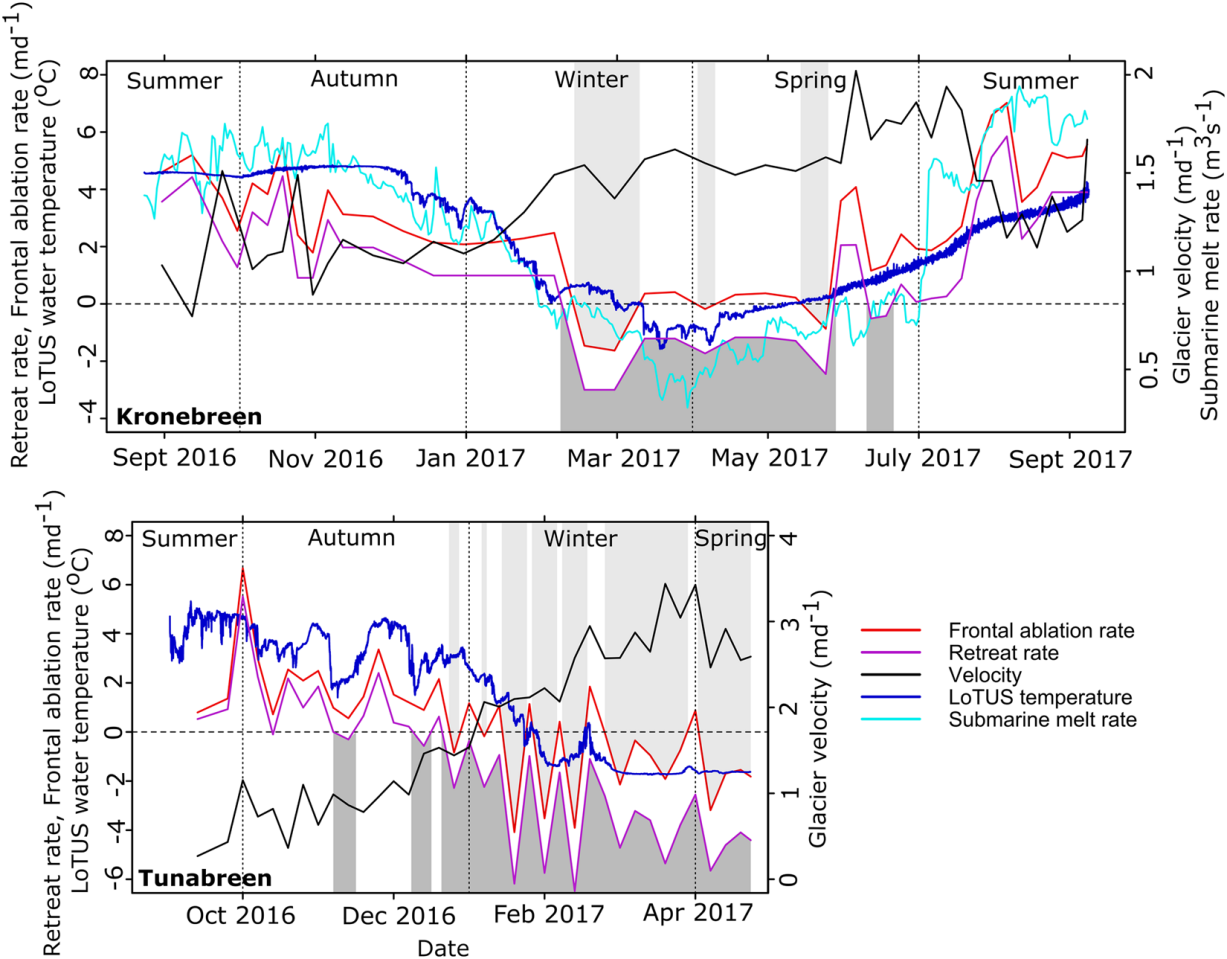
The frontal ablation rate, and its constituent variables retreat rate and velocity, are shown for both Kronebreen (top) and Tunabreen (bottom). The light grey areas highlight areas of ‘frontal accumulation’, as calculated by Eq. 1, and the dark grey areas highlight areas of margin advance. Additionally, the water temperature at depth from the LoTUS buoys in each fjord, and the calculated submarine melt rate for Kronebreen are shown. The plots have different scales, but share the same horizontal axis. Plots created in R^36^.

*U*_*T*_ is terminus ice speed (*md*^−1^) and $\frac{dl}{dt}$ is the change in ice front position with time (*md*^−1^), with positive/negative values corresponding to advance/retreat. Figure 3 shows both glacier’s frontal ablation rates, as well as the two constituent components of terminus speed and frontal advance/retreat rate. Ocean temperature at depth, harvested from the LoTUS buoys (see Methods) in close proximity to the front of the glaciers, is plotted along with the glacier dynamic variables.

In Kongsfjorden, Kronebreen’s terminus retreats over most of the observation period, with the rate sometimes exceeding 5 *md*^−1^. However, the terminus instead advances between February and June 2017, with the greatest rate of advance being around 3 *md*^−1^. Overall, during the whole study period (25 August 2016 to 4 September 2017), Kronebreen retreats an average of 1.03  *md*^−1^ (396.8 *m* in total), comparable to values of annual retreat previously reported^25^. Terminus velocity at Kronebreen exhibits min/max values of 0.8 *md*^−1^ and 2 *md*^−1^ in Autumn 2016 and early Summer 2017 respectively. Velocities are consistently higher towards the centre of the glacier, reaching nearly 3.5 *md*^−1^ in July 2017. Figure 3 shows that the time series of frontal ablation resembles the time series of the retreat rate more closely than that of velocity. Calculated frontal ablation is negative for a few short periods, most notably February–March 2017. This occurs when measurements indicate the glacier is advancing at a greater rate than the velocity, rather than being indicative of ‘frontal accumulation’. For Kongsfjorden, the lowest fjord water temperature recorded is −1.58 °C on 18 March 2017, and the highest is 4.85 °C on 8 November 2016. During most of the time series, low frequency variations are apparent, but from Spring 2017 onwards, the time series instead exhibits high frequency, low-magnitude oscillations superimposed on a general warming trend. The average fjord water temperature recorded by the LoTUS buoy is 2.23 °C.

**Figure 4:**
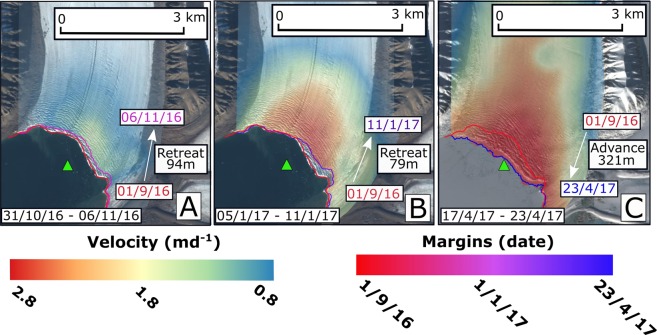
Velocity fields for Tunabreen calculated for the period shown in the bottom left corner of each panel are displayed. Margin positions from 1 September 2016 and the end of the velocity field calculation period are also shown, along with the mean value across the glacier front of retreat/advance between these two dates. The position of the LoTUS buoy is portrayed by a green triangle. The background images for these panels are from 9 September 2016 (**A**,**B**) and 6 April 2017 (**C**). The background images are Copernicus Sentinel data (2016, 2017) downloaded from the Copernicus Open Access Hub (https://scihub.copernicus.eu/). Terms of use available at https://scihub.copernicus.eu/twiki/do/view/SciHubWebPortal/TermsConditions.

In Tempelfjorden, during the period 2 September 2016 until mid December 2016, Tunabreen exhibits slow velocities and margin retreat. However, from mid December 2016 until the end of the study period (21 April 2017), Tunabreen’s margin re-advances to approx. 320 *m* in front of its location at the beginning of September 2016. This was accompanied by an increase in frontal velocities, with average frontal velocities increasing from <1 *md*^−1^ in September 2016 to >3 *md*^−1^ in March 2017. Examples of velocity fields and margin positions from different dates during the study period are shown in Fig. 4, and suggest that the glacier was surging. This is important to note as it means that the dynamic behaviour of Tunabreen was atypical during our study period. The pattern of frontal ablation followed the pattern of frontal advance/retreat more closely than the velocity variations, similarly to Kronebreen. The subsurface temperatures in Tempelfjorden were, on average, cooler than those measured in Kongsfjorden. The mean LoTUS temperature was 1.5 °C, with a minimum of −1.74 °C and a maximum of 5.33 °C.

### Regression analysis

The possible drivers of frontal ablation that we analysed in this study are fjord water temperature at depth, sea surface temperature, air temperature, precipitation, and sea ice cover.

A simple linear regression analysis (see Methods) between each of these explanatory variables and the satellite derived frontal ablation returns the following *R*^2^ values for Kronebreen: 0.47 (water temperature at 67 *m* depth), 0.46 (sea surface temperature), 0.37 (air temperature), and 0.1 (sea ice). Precipitation was not significant on the 95% level and thus not relevant as a predictor. This means that water temperature at depth is the strongest predictor of frontal ablation rate, closely followed by sea surface temperature. In a multiple linear regression, water temperature at depth, sea surface temperature, and air temperature were significant at the 95% level with regards to the Kronebreen data set, rendering an *R*^2^ value of 0.6699, and an ${\overline{R}}^{2}$ value, which is preferred, of 0.667 (see methods for explanation). The inclusion of two extra predictors on top of water temperature at depth thus accounts for an additional 20% of Kronebreen’s behaviour. The three significant variables from the multiple linear regression are plotted alongside each other in Supplementary Fig. S3.

The following *R*^2^ values were obtained from simple linear regression analysis focusing on Tunabreen: 0.45 (water temperature at 19 *m* depth), 0.4 (sea surface temperature), 0.24 (sea ice), 0.2 (air temperature). Precipitation was not significant on the 95% level for Tunabreen and thus not relevant as a predictor. Water temperature at depth was, as for Kronebreen, the strongest predictor of frontal ablation. In a multiple linear regression, only water temperature at depth and sea surface temperature were significant at the 95% level and yielded an ${\overline{R}}^{2}$ value of 0.47. Thus, the inclusion of an extra predictor on top of water temperature at depth only accounts for an extra 0.02% of frontal ablation at Tunabreen.

### Submarine melt rate

The rate of frontal ablation is based on glacier front changes observed from satellites, and thus only captures changes occurring above the waterline. Hence, the frontal ablation rate does not explicitly account for submarine processes such as submarine melt or subaqueous calving^26^. Thus, a submarine melt rate *Q*_*m*_ for the terminus of Kronebreen has been calculated using a parametrization^27^ based on local fjord water temperatures and salinities (see Eqs 2–4, Methods section). The submarine melt rate has been found to account for, on average, 25.6% of the frontal ablation rate in terms of volume (see Fig. 3). As a result of there being no data available of water current velocities, this parameterisation does not include any melting from plumes, despite evidence of plumes at Kronebreen^28^ and Tunabreen^17^ As such, it is likely to be an underestimation of the submarine melt rate.

Due to a lack of salinity data from Tempelfjorden, as well as the truncated time series of water temperatures, no submarine melt rate was calculated for Tunabreen.

## Discussion

The data from the SVPs provide clear evidence for the presence of AW in close proximity to Kronebreen during August 2016 (see Fig. 2). In addition to this, the warm water temperatures (>3 °C) measured by the Kongsfjorden LoTUS buoy at various points during Summer/Autumn 2016 and Summer 2016 strongly suggest that AW intrusion occurred during elongated periods of time. The Tempelfjorden LoTUS also recorded temperatures in excess of >3 °C during Summer and Autumn 2016, which are likely to be indicative of AW. When considering this data alongside the results presented by Prominska *et al*.^23^ (not available for Tempelfjorden), it appears that whilst there was no evidence for AW intruding into the inner part of Kongsfjorden during the period 2001–2013, there is strong evidence for the presence of AW in close proximity to Kronebreen during both 2014 and 2016. This suggests that AW has only been intruding into the inner part of Kongsfjorden during recent years, and thus provides observational evidence for the Atlantification of water masses in Kongsfjorden. Although there is no historical data for Tempelfjorden, and so no possibility to look at temporal changes in AW intrusion, the results presented here provide evidence that, at least during late 2016, warm waters were present in the innermost part of the fjord.

The results presented by Prominska *et al*.^23^ provide evidence for the water masses in the inner and outer parts of Kongsfjorden being, in most years, dissimilar. An example of this is presented in Fig. 2, where an adaptation of the data from 2013 is shown. Here, it can be seen that AW was present in various parts of the outer part of Kongsfjorden, but that AW is absent in the inner fjord. Instead, local water (temperatures of <1 °C) is instead found at depth in the inner fjord. Therefore, measurements of water properties taken in the outer part of the fjord during this time are unlikely to be representative of conditions near the glacier front. The fact that dissimilar water masses have been recorded in the inner and outer parts of Kongsfjorden during many different years (see Prominska *et al*.^23^) suggests that, when relating oceanographic properties to glacier behaviour, it is important to sample water masses from as close to glaciers as possible. It must, however, be noted that all the the oceanographic measurements used to create the water mass plots were taken during summer periods, and there may be some seasonal variation in this pattern.

The results from the regression analysis show that, for both Kronebreen and Tunabreen, subsurface ocean temperatures can explain the greatest proportion of variation in the rates of frontal ablation. This therefore provides evidence for ocean temperatures being of importance for tidewater glaciers in Svalbard, agreeing with previous results^2^. This is further compounded by considering the results of the submarine melt calculation, where the magnitude of the (conservative) submarine melt rate at Kronebreen was found to be significant with relation to the frontal ablation rate. These results show that the ocean temperatures are of importance for frontal ablation and thus that the Atlantification of waters around Svalbard is having an impact on the mass balance of tidewater glaciers. However, other variables such as air temperature, sea surface temperature, and sea ice concentration were also found, via the regression analysis, to exert a significant influence on frontal ablation. It is thus important to consider both atmospheric and oceanographic variables when trying to explain changes in glaciological behaviour. This supports the results from a recent study which, although focusing on a different area, found that tidewater glacier retreat on Greenland’s East coast can be explained by considering combined atmospheric and oceanic conditions^29^. Although it appears likely that further Atlantification will increase frontal ablation rates, the influence of site specifc factors such as bathymetry means that glaciers may react heterogoneously to current and future changes, and care should be taken when upscaling results.

Despite the evidence for the strong influence of subsurface temperatures on frontal ablation, this impact is likely to vary with temperature values. High water temperatures significantly impact frontal ablation rates through causing increased submarine melt which, in turn, can promote calving. However, when water temperatures are low, other factors such as velocity gradients or air temperature may exert a greater control. As there is evidence that low subsurface temperatures can occur at glacier fronts whilst warm AW is present further away from the glacier, the distance of water temperature measurements from the glacier is of consequence when assessing the importance of subsurface water temperatures on frontal ablation.

Although the finding that frontal ablation rates are greatly influenced by ocean temperatures at depth corresponds to the conclusions of a previous study by Luckman *et al*.^2^, there are some important differences. In both cases, frontal ablation was calculated using the same equation, and a simple linear regression was conducted between the frontal ablation rate and subsurface ocean temperatures. However, whilst the *R*^2^ values from this study are 0.47 for Kronebreen and 0.45 for Tunabreen, the values for the Luckman *et al*.^2^ study are 0.84 for Kronebreen and 0.8 for Tunabreen.

The first possible explanation for this disparity is inter-annual variation, with the Luckman *et al*.^2^ study using data from January 2013 until July 2014, whilst this study looks at the period August 2016 until September 2017 (Kronebreen) and September 2016 until April 2017 (Tunabreen). Taking a 10 month period (January until September) covered by both Kronebreen studies, frontal retreat is found to be ~200 to 250 *m* in 2016–2017, but was instead around 300 *m* in 2013–2014. During 2013–2014, velocities for Kronebreen reported by Luckman *et al*.^2^, peak at ~4 *md*^−1^, which is higher than the peak of ~2 *md*^−1^ in 2016–2017. For Tunabreen, the occurrence of a surge during 2016–2017 (see Fig. 4) means that the glacier was exhibiting very different dynamic behaviour during the two study periods. There is therefore clear evidence for a degree of inter-annual variation, which is highly likely to be responsible for some of the differences in results between the two studies.

However, it is also important to note that the moorings from which ocean temperatures were measured by Luckman *et al*.^2^ were located much further away from the glacier than the ones used for this study (see Fig. 1). Considering this in conjunction with the aforementioned differences in water properties between the inner and outer parts of Kongsfjorden raises the possibility that the temperatures measured by Luckman *et al*.^2^ are not representative of those coming into contact with the glacier. This may have led to the impact of ocean temperatures on frontal ablation being misestimated. As a result of this, it can be seen that measuring water temperatures from as close to the calving front of glaciers as possible is important in preventing the inclusion of additional uncertainty into studies focused on frontal ablation rates.

Another aspect to consider when comparing the results of this study with those reported by Luckman *et al*.^2^ is the difference in how temperature is actually measured: During 2016–2017, water temperature was measured from a single point below the surface. In contrast, Luckman *et al*.^2^ used a single figure for multiple, averaged water temperatures between 20 *m* and 60 *m* depth. Differences in temperature with depth may be significant, and likely to be more pronounced during summer when the fjords are more stratified as a result of freshwater influx^30^. For this reason, an array of temperature sensors at different depths is preferable and should be implemented where possible.

Finally, it is also important to note that the length of the study period between the two studies is a different. It may be the case that there is a seasonal signal in the relationship between subsurface water temperatures and frontal ablation rates, and that the inclusion of two winter periods in the 2013–2014 study, compared to just one in the 2016–2017 study, can explain some of the discrepancy in the results.

## Concluding Remarks

This study adds to a growing body of evidence that shows ocean temperatures at depth to be of a dominant importance for the frontal ablation rate of tidewater glaciers. However, the use of a novel glacier-proximal data set has revealed that this relationship is not as strong as previously reported, at least for Kronebreen and Tunabreen. The complex interactions between a number of different oceanographic, topographical, glaciological, and meteorological factors mean that each tidewater glacier is exposed to, and responds differently to, external forcings. The result of these intricacies is that uncertainties and unknowns surrounding the topic of ice-ocean interactions are rife, and uncertainties in estimates of future trends are further compounded by the poor or non-existent representation of some calving processes in numerical models^31^. Consequently, it is imperative to sample water temperatures from as close to glacier termini as possible in order to prevent the inclusion of additional uncertainty in estimates of both present and future frontal ablation rates.

## Methods

### Satellite derived frontal ablation rate

It is assumed that frontal ablation is described by Eq. 1. As only measurements from above the waterline enter 1, $\dot{a}$ has previously been interpreted to represent the calving rate instead of frontal ablation rate^26,32^. Frontal ablation is comprised of surface velocity at the glacier terminus, and changes of the terminus over time (advance/retreat rate).

Surface velocities were acquired by using the method of offset tracking on pairs of orbitally corrected Sentinel 1 GRDH images co-registered using the ACE30 DEM. The time between image acquistions was not constant throughout the study period but was always a multiple of 6 days. Some error is included in the velocity calculations, and can be estimated by considering the presence of movement vectors across stable areas such as rocky outcrops. For both glaciers, this was low with values of around 0.3 *md*^−1^ being measured in these areas. Examples of the velocity fields can be seen in Fig. 4. The orientations of the velocity vectors were also consulted to confirm that the calculated velocities were in the expected direction. A land-sea mask was applied to counter the effects of changing sea ice conditions in the terminal region being recorded as glacier velocity. Margin positions for Kronebreen were manually digitised using orbitally and terrain corrected (range-doppler method) Sentintel 1 GRDH images. Locations where digitisation was difficult due to the presence of sea ice, which looks similar to glacier ice on the images, were excluded to reduce error. However, some digitisation error is likely to remain. All the satellite images used were from the same track. Velocity and margin change measurements for Kronebreen were extracted in QGIS at flowlines equally spaced every 250 *m* across the glacier, with the mean value of all the flowlines used to represent the relevant period. The velocity measurements were taken at a fluxgate at 12.65°E, which is close to the terminus but at a location where the velocity fields spanned the entire width of the glacier.

For Tunabreen, velocity measurements were also harvested in QGIS but margin change was instead quantified using MaQiT^33^.

### Water mass characterization in the fjord(s)

The water masses in Kongsfjorden were characterized based on 11 sound velocity profiles taken on the 23rd to the 26th August 2016 in close proximity to the terminus of Kronebreen. The location of the sound velocity profiles (SVPs) is shown in Fig. 2. A twelfth SVP profile was taken at a location further away from the glacier, and so was not used to create the plot of water masses in Fig. 2. Data from this twelfth profile is, however, still shown alongside the other eleven profiles in Supplementary Material Fig. S2. The data was collected using a Valeport miniSVP, which includes a sound velocity sensor, and temperature sensor, and a pressure transducer. The raw data collected by this instrument thus consists of temperature, pressure, and sound velocity. Valeport Datalog X2 software was used to extract salinity and depth measurements from the downward profiles using the method/relationships described by Allen *et al*.^34^. Approximately the top 70 *cm* of the profiles were discarded to ensure that all the values considered were measured after the sensor had acclimatised. The salinity and temperature values at each location were then used to classify the water mass in accordance with the following criteria^23^; Atlantic water (>3 °C, >34.9 *psu*), transformed atlantic water (>1 °C, >34.7 *psu* but cooler and less saline than AW), surface water (>1°C, <34 *psu*), and intermediate water (>1 °C, 34 to 34.7 *psu*). The sound velocity probes revealed that large part of inner Kongsfjorden, including terminus proximal areas, held water masses warmer than 3 °C and with salinities greater than 34.9 *psu*, thus, Atlantic water^11,12,23,35^. In order to display the data graphically, all the SVP profiles were plotted according to only to their longitude and superimposed onto the bathymetric transit shown in Fig. 2.

Panels A and B from Fig. 2 are an illustration of data from Prominska *et al*.^23^, collected during the summers of 2013 and 2014.

### Ocean temperature at depth from Long-Term Underwater Sensing Bottom Landers (LoTUS)

**Lo**ng **T**erm **U**nderwater **S**ensing bottom landers (LoTUS buoys) are non-commercial instruments developed at KTH Royal Institute of Technology, Stockholm, and are here used to measure ocean temperature at a pre-defined frequency of every 10 minutes. As LoTUS buoys can be deployed instantaneously at any given location, and from any given platform (at sea or in the air), they are ideally suited to be moored in close proximity to calving glacier fronts.

The Kongsfjorden LoTUS was moored 15 *m* above the sea floor, translating into 67 *m* below average sea level (±1 m due to tidal variations) during the period 25 August 2016 and 4th Sept 2017. During this period, LoTUS collected more than 54 000 samples, and the total time drift of the instrument was 54 min 55 sec. The raw temperature data was corrected for the time drift. A Star-Oddi CTD DST sensor, which is unrelated to the LoTUS technology, was attached to the LoTUS buoy in Kongsfjorden, allowing for the temperatures recorded by the two independant devices to be compared. The two datasets showed excellent correspondence, with both instruments showing the high frequency, low magnitude oscillations at the beginning of the study period. Depth data for the whole study period was also available from the Star-Oddi sensor, as well as a truncated salinity time series most likely cut short due to biofouling and therefore of no use for the calculation of submarine melt.

The Tempelfjorden LoTUS was located 35 *m* above the sea floor, which meant it was at an average depth of 19 *m* (±1 m due to tidal variations). The LoTUS transmitted 37 920 data points, of which 33 246 where used. This is due to the fact that temperature recording began prior to deployment, and only temperatures measured after the buoy was in place and acclimatised were used.

### Regression analysis, utilising meteorological variables and sea-surface data

Five environmental variables were chosen as possible predictors of frontal ablation. Water temperature at depth from the LoTUS buoy, sea surface temperature, air temperature, precipitation, and sea ice cover. Precipitation (daily totals) and air temperature (daily means) for Kronebreen/ Kongsfjorden were obtained from the Norwegian Meteorological Institute using the eKlima service (www.eklima.met.no). The data is from the nearest meteorological station, which is the Ny Ålesund observation site. Sea ice data is from the Norwegian Ice Service (http://polarview.met.no/) where the ice cover is classified manually according to internationally recognised criteria. Sea surface temperature data was collected as a daily NetCDF product from the Multi-scale Ultra-high resolution (MUR) Sea Surface Temperature (SST) data set (https://mur.jpl.nasa.gov, a MEaSUREs dataset), which provides global sea surface temperature data as a spatial resolution of 0.01 degrees latitude by 0.01 degrees longitude (roughly 1 *km* by 1 *km*). The time series is a blend of infrared sensor data (from MODIS and AVHRR) and microwave data (from AMSR-E). The daily SST value from as close to Kronebreen as possible was extracted from the downloaded files using Matlab. The regression analysis for this study was carried out using the software *R*^36^. Before analysis began, all of the time series were re-sampled to give daily values. Days with missing data were left blank. Results from the regression analyses are only presented if they had a *p* value of 0.05 or lower. For the multiple linear regression analysis, the VIF (variance inflation factor) was also consulted with variables where the square root of the VIF was higher than 2 being excluded. Additionally, it was the ${\overline{R}}^{2}$ value that was consulted for the multiple linear regression as opposed to the *R*^2^ value, which was used for the simple linear regression. This is because the ${\overline{R}}^{2}$ value is adjusted for the number of predictors in the model, only increasing if the new predictor improves the model more than would be expected by chance. The importance of lagged variables was also investigated. To do this, the CCF (cross correlation function) values of each explanatory variable were used to identify the possible number of lags which were of importance. As the data represents time series, only negative lags (those going back in time) were of interest. The regression was then re-performed including the possible lagged variables, but none had a satisfactory *p* value.

The comparison made between the *R*^2^ values presented in this paper with those presented by Luckman *et al*.^2^ is based on the results of the simple linear regression between frontal ablation and subsurface water temperature. Both studies calculated frontal ablation using the same calculation. The methodology employed to calculate the values by the two studies is thus the same, meaning a valid comparison can be made.

### Submarine melt rate and classification of water masses

Using the temperature data from the LoTUS buoys, as well as a representative value for salinity in Kongsfjorden, an estimate of the submarine melt rate for Kronebreen was calculated from the following parameterisation^27^:

2 $${Q}_{m}={Q}_{h}/({\rho }_{i}{L}_{i})$$

3 $${Q}_{h}={\rho }_{w}{C}_{pw}{\gamma }_{T}({T}_{ocean}-{T}_{f}){A}_{eff}$$

4 $${T}_{f}=0.0939-0.057S+7.64\times {10}^{-4}z$$

here, *Q*_*m*_ is melt rate (*m*^3^ *s*^−1^), *Q*_*h*_ is heat flux (*Js*^−1^), *ρ*_*i*_ is the density of ice (*kg m*^−3^), *L*_*i*_ is the latent heat of fusion for ice (*J kg*^−1^), *ρ*_*w*_ is the density of freshwater (*kg m*^−3^), *C*_*pw*_ is the specific heat capacity of water (*J* (*kg*)^−1^), *γ*_*T*_ is the thermal conductivity of water (*m s*^−1^), *T*_*ocean*_ is ocean temperature (°C), *T*_*f*_ is freezing point temperature (°C) at a given depth (*z*, given in *m*) and salinity (*S*, given in *psu*), and *A*_*eff*_ is the area over which melting occurs (*m*^2^). For *ρ*_*i*_, *L*_*i*_, *ρ*_*w*_, *C*_*pw*_, *γ*_*T*_, the same values were chosen as in the original study^27^. In addition to these, a value for the area over which melting occurs (*A*_*eff*_) was needed. This area was calculated by multiplying the length of the glacier as measured using Google Earth (3600 *m*) with a representative value for the underwater height of the glacier (60 *m*, chosen after consulting the bathymetry of Kongsfjorden from as close to the glacier front as possible). The resulting value of 216000 *m*^2^ was split in two as the calculation was done for the ‘top’ and ‘bottom’ halves of the glacier front using two different values for ocean temperature (*T*_*ocean*_) and depth (*z*). The two different *T*_*ocean*_ values were the sea surface temperature and the temperature from the LoTUS buoy, and the *z* value corresponded to the point where these temperatures were recorded (0 *m* and 67 *m* respectively). No salinity time series from the proximal area of Kronebreen spanning the entire LoTUS time period was available. The salinity value (*S*) was thus kept constant and derived from taking the mean of a salinity time series collected further out in Kongsfjorden. The results from the top and bottom halves of the glacier were then added together to give one value for submarine melt across the whole glacier. This simple parameterisation was chosen instead of a more complex one as the inputs required for the calculation match with the available data. Specifically, the lack of any data relating to water currents (direction or velocity) meant that a plume paramterisation was not possible^37^.

## Supplementary information

Supplementary Material: Relating ocean temperatures to frontal ablation rates at Svalbard tidewater glaciers: Insights from glacier proximal datasets

## Data Availability

The meteorological data (air temperature, precipitation) used for this study is publicly available using the eKlima service from the Norwegian Meteorological Institute (http://eklima.met.no/). Sea ice data is available via the Sea ice Service from the Norwegian Meteorological Institute (http://polarview.met.no/). The satellite data used to collect velocities and margin change data is freely available from the Copernicus open access hub (https://scihub.copernicus.eu/). The work flows used to evaluate margin change and velocities are detailed in this paper, but more information on the offset tracking method used to calculate velocities is available from the ESA website (http://step.esa.int/main/doc/tutorials/). Sea surface temperature data is available from the podacc/JPL website (https://podaac.jpl.nasa.gov/Multi-scale_Ultra-high_Resolution_MUR-SST). The subsurface water temperatures collected from this study will shortly be available on the Bolin Centre database (https://bolin.su.se/data/).
