# Supplementary material for: Relating ocean temperatures to frontal ablation rates at Svalbard tidewater glaciers: Insights from glacier proximal datasets
Source: Sci Rep. 2019 Jul 1;9:9442. doi: 10.1038/s41598-019-45077-3 (PMC6603002; doi:10.1038/s41598-019-45077-3)
Supplement: Supplementary file 1 — Supplementary Material: Relating ocean temperatures to frontal ablation rates at Svalbard tidewater glaciers: Insights from glacier proximal datasets [file 41598_2019_45077_MOESM1_ESM.pdf]

Supplementary Material

Relating ocean temperatures to frontal ablation rates at Svalbard tidewater glaciers: Insights from glacier proximal datasets

Felicity A. Holmes, Nina Kirchner, Jakob Kuttenkeuler, Jari Krützfeldt, and Riko Noormets

S1: Sound velocity profiles

Eleven sound velocity profiles were taken at various glacier- proximal locations in Kongsfjorden during August 2016, and one was taken further away from the glacier (profile A). The locations of all the profiles are shown on Supplementary Fig. 1. Both temperature and salinity with depth was available from these profiles (see Methods), which are plotted in supplementary Fig.2. Changes in temperature were often accompanied by changes in salinity. For all but three of the profiles (A, G, and L), the highest temperatures were found at depths of between 20 and 25 m.

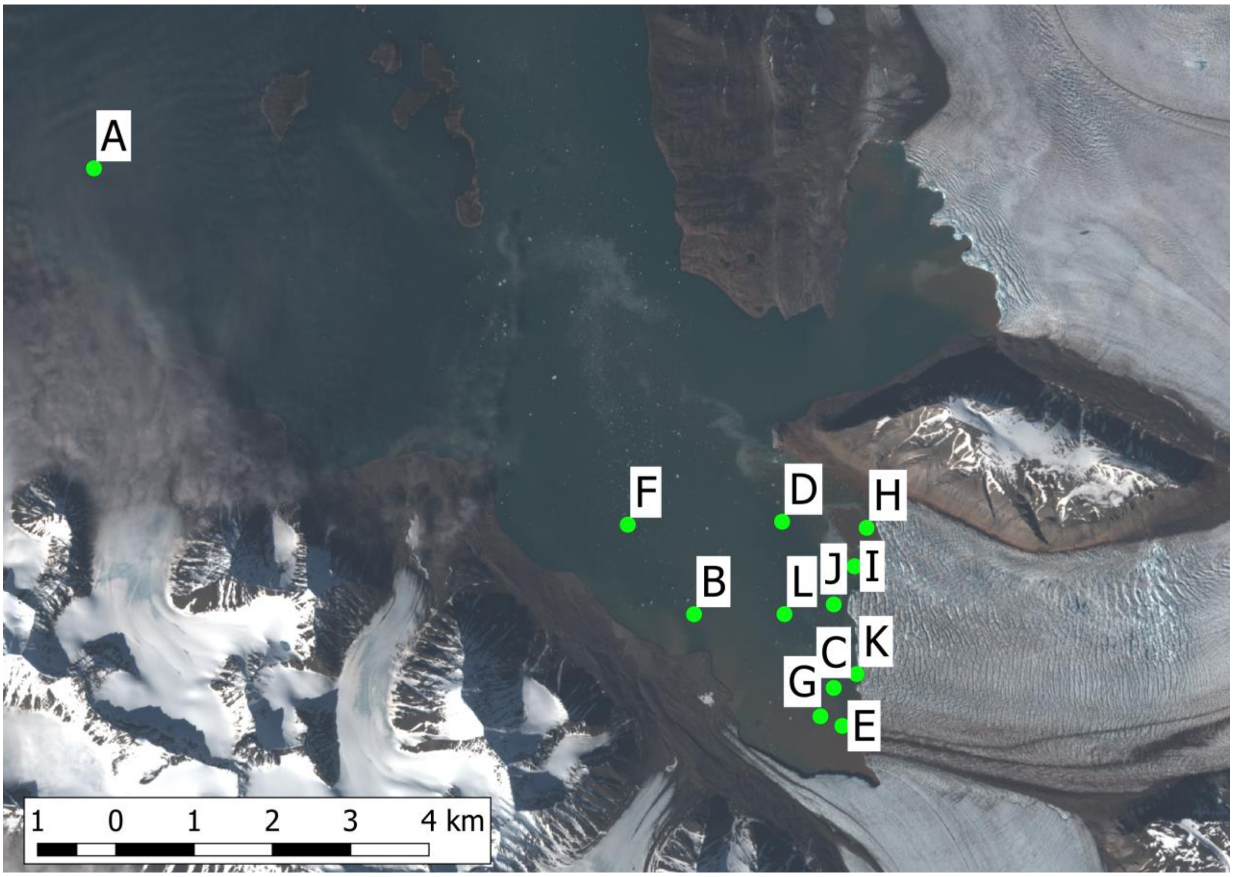

Supplementary Figure S 1: Map of Kongsfjorden showing the locations of each of the SVP profiles. The background image is Copernicus Sentinel data (2016) downloaded from the Copernicus Open Access Hub (https://scihub.copernicus.eu/). Terms of use available at https://scihub.copernicus.eu/twiki/do/view/SciHubWebPortal/TermsConditions.

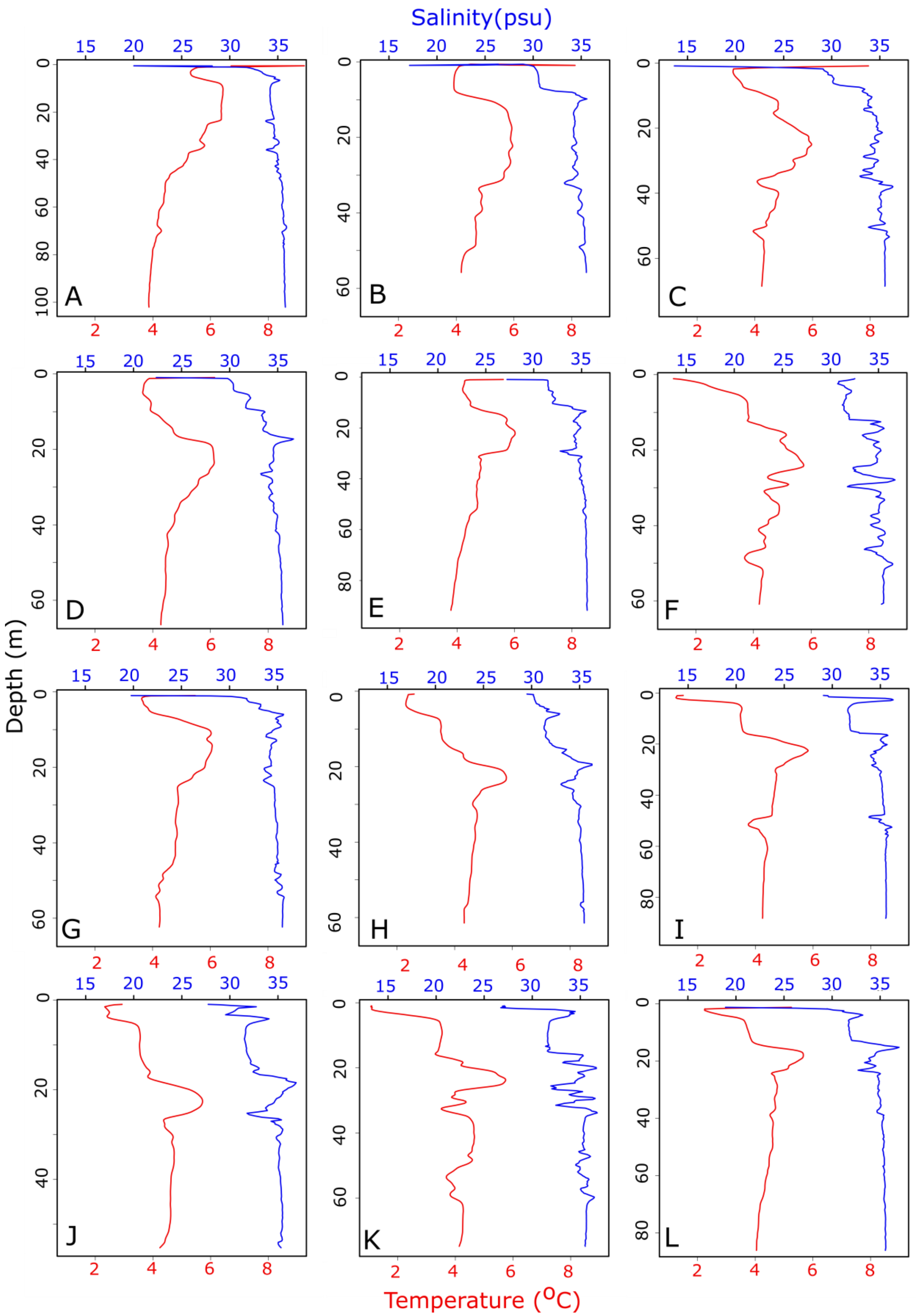

Supplementary Figure S 2: Salinity and temperature with depth for each of the SVP profiles. X axes scales are the same for all panels, but the y axis (depth) varies. Plots created in R.

S2: Temperature(s) time series

For the multiple linear regression, air temperature, sea surface temperature, and sub-surface water temperature were all significant (for Kronebreen). These variables are plotted alongside eachother below, for both glacier-fjord settings. The plots show subsurface water temperatures to be warmer than sea surface temperatures during the period September 2016 – late January 2017.

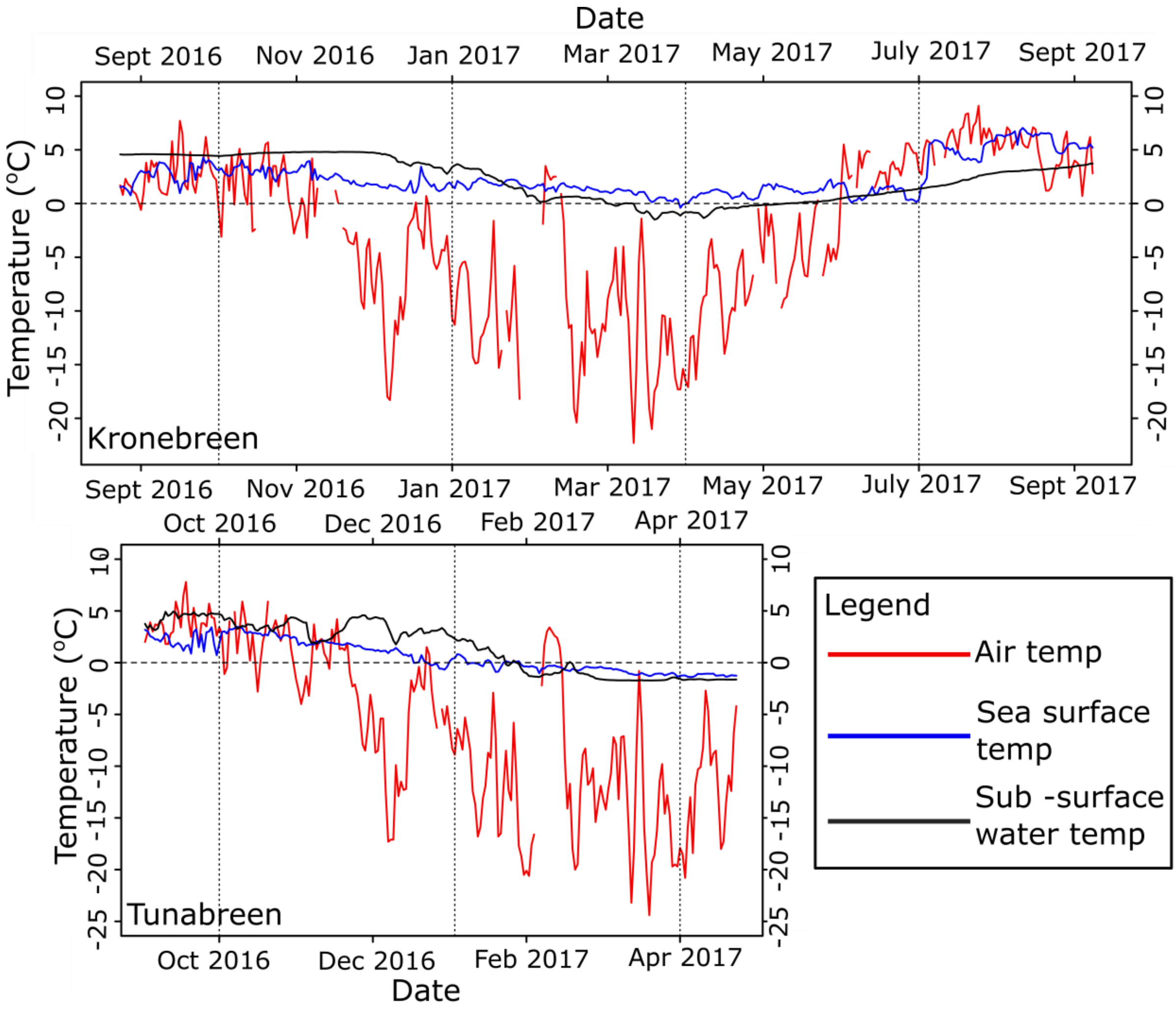

Supplementary Figure S 3:Time series of air temperature, sea surface temperature, and water temperature at depth plotted alongside each other for both Kronebreen and Tunabreen. The plots share an x axis, but have distinct y axes. The air temperature data is from the Norwegian Meteorological Institute, the Sea surface temperature data from the Multi-scale Ultra-high resolution (MUR) Sea Surface Temperature (SST) data set (https://mur.jpl.nasa.gov, a MEaSUREs dataset), and the subsurface water temperature from the LoTUS buoy deployed for this study. Plot created in R.
